# Clinical performance and patient outcome after simulation-based training in prevention and management of postpartum haemorrhage: an educational intervention study in a low-resource setting

**DOI:** 10.1186/s12884-017-1481-7

**Published:** 2017-09-11

**Authors:** Ellen Nelissen, Hege Ersdal, Estomih Mduma, Bjørg Evjen-Olsen, Jos Twisk, Jacqueline Broerse, Jos van Roosmalen, Jelle Stekelenburg

**Affiliations:** 1 Research Department, Haydom Lutheran Hospital, POB 9000, Haydom, Manyara Tanzania; 2 Department of Obstetrics and Gynaecology, Southmead Hospital, Southmead Road, Bristol, BS10 5NB UK; 3 Stavanger Acute Medicine Foundation for Education and Research (SAFER), Department of Anaesthesiology and Intensive Care, Stavanger University Hospital, POB 8100, 4068 Stavanger, Norway; 4 Centre for International Health, University of Bergen, Årstadveien 21, N-5009 Bergen, Norway; 5 Department of Obstetrics and Gynaecology, Sørlandet Hospital, Engvald Hansens vei 6, 4400 Flekkefjord, Norway; 6 Department of Epidemiology and Biostatistics, VU University Medical Center, POB 7057, 1007 MB, Amsterdam, The Netherlands; 7 Faculty of Earth and Life Sciences, Department of Methodology and Applied Biostatistics, VU University Amsterdam, de Boelelaan 1085, 1081 HV, Amsterdam, The Netherlands; 8 Athena Institute, Faculty of Earth and Life Sciences, VU University Amsterdam, de Boelelaan 1085, 1081 HV, Amsterdam, The Netherlands; 9 Department of Obstetrics, Leiden University Medical Centre, Albinusdreef 2, 2333 ZA Leiden, The Netherlands; 10 Department of Obstetrics and Gynaecology, Leeuwarden Medical Centre, Henri Dunantweg 2, 8934 AD, Leeuwarden, The Netherlands; 11 Department of Health Sciences, Global Health, University Medical Centre Groningen/University of Groningen, Antonius Deusinglaan 1, 9713 AV, Groningen, The Netherlands

**Keywords:** Obstetrics, Simulation-based training, Postpartum haemorrhage, Low-resource settings, Education

## Abstract

**Background:**

Postpartum haemorrhage (PPH) is a major cause of maternal mortality. Prevention and adequate treatment are therefore important. However, most births in low-resource settings are not attended by skilled providers, and knowledge and skills of healthcare workers that are available are low. Simulation-based training effectively improves knowledge and simulated skills, but the effectiveness of training on clinical behaviour and patient outcome is not yet fully understood. The aim of this study was to assess the effect of obstetric simulation-based training on the incidence of PPH and clinical performance of basic delivery skills and management of PPH.

**Methods:**

A prospective educational intervention study was performed in a rural referral hospital in Tanzania. Sixteen research assistants observed all births with a gestational age of more than 28 weeks from May 2011 to June 2013. In March 2012 a half-day obstetric simulation-based training in management of PPH was introduced. Observations before and after training were compared. The main outcome measures were incidence of PPH (500–1000 ml and >1000 ml), use and timing of administration of uterotonic drugs, removal of placenta by controlled cord traction, uterine massage, examination of the placenta, management of PPH (>500 ml), and maternal and neonatal mortality at 24 h.

**Results:**

Three thousand six hundred twenty two births before and 5824 births after intervention were included. The incidence of PPH (500–1000 ml) significantly reduced from 2.1% to 1.3% after training (effect size Cohen’s d = 0.07). The proportion of women that received oxytocin (87.8%), removal of placenta by controlled cord traction (96.5%), and uterine massage after birth (93.0%) significantly increased after training (to 91.7%, 98.8%, 99.0% respectively). The proportion of women who received oxytocin as part of management of PPH increased significantly (before training 43.0%, after training 61.2%). Other skills in management of PPH improved (uterine massage, examination of birth canal, bimanual uterine compression), but these were not statistically significant.

**Conclusions:**

The introduction of obstetric simulation-based training was associated with a 38% reduction in incidence of PPH and improved clinical performance of basic delivery skills and management of PPH.

## Background

Postpartum haemorrhage is a major cause of maternal mortality. [1] The prevalence of postpartum haemorrhage ranges from 11% to 26% for blood loss more than 500 ml, and from 2 to 5% for blood loss more than 1000 ml. [2, 3] More than 55% of pregnant women in Africa suffer from anaemia in pregnancy, [4] and do not have much reserve when postpartum haemorrhage occurs. Prevention and prompt, adequate treatment of postpartum haemorrhage is therefore important. However, many births in sub-Sahara Africa are not attended by skilled providers. [5] In Tanzania only 51% of all births are assisted by a skilled provider. [6] In addition, knowledge and skills of providers are low [5, 7] and in-house training facilities to keep knowledge and skills of healthcare workers up to date hardly exist in low-resource settings. [5, 8] Therefore, Jhpiego (John Hopkins Programme for International Education in Gynaecology and Obstetrics) and Laerdal Global Health developed Helping Mothers Survive Bleeding After Birth (HMS BAB), a simulation-based training package targeted at healthcare workers in areas with a high burden of maternal morbidity and mortality. [9] The training package focuses on basic delivery care, active management of third stage of labour, and treatment of postpartum haemorrhage. [10] Simulation-based education, and HMS BAB in specific, has been shown to effectively increase knowledge, skills, and confidence of healthcare workers [8, 11–13] and is intended to reduce maternal morbidity and mortality caused by postpartum haemorrhage.

**Fig. 1:**
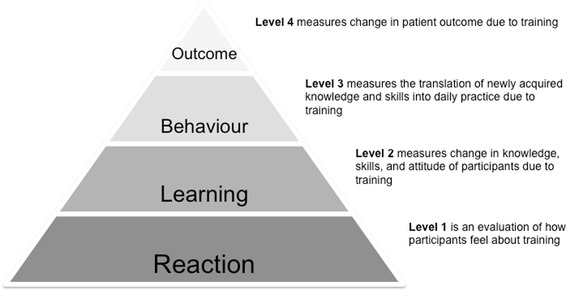
Kirkpatrick model for evaluating training programmes

The four-level Kirkpatrick model is often used to evaluate training programmes (Fig. 1). [14] Most publications regarding evaluation of training programmes only address the first two levels of this model. [12, 15] Improvement in clinical performance of healthcare workers and patient outcome is the ultimate goal, however most research in this area has been limited to neonatal outcome. [16, 17] There is mounting evidence to suggest that training in emergency obstetric care improves maternal outcome. [18–22]

The aim of this study was to assess the effect of obstetric simulation-based training on the incidence of postpartum haemorrhage (Kirkpatrick level 4) and clinical performance of basic delivery skills and management of postpartum haemorrhage (Kirkpatrick level 3) in a low-resource setting.

## Methods

### Study design and setting

We conducted a prospective educational intervention study at Haydom Lutheran Hospital, a rural referral hospital in Northern Tanzania. During the study period, the hospital had 420 beds and provided free reproductive health services including comprehensive emergency obstetric care. This incorporated facilities for basic delivery (including the provision of antibiotics, uterotonics, and anticonvulsants), assisted vaginal delivery, removal of retained products of conception, blood transfusion, and caesarean section. Extrapolating from the 2002 census, the immediate catchment area of the hospital covered a population of approximately 350,000 in 2012, while the greater reference area covered a population of approximately 2,357,000. [23] The annual number of hospital-based births at the time of the study was approximately 5000.

### Intervention

**Fig. 2:**
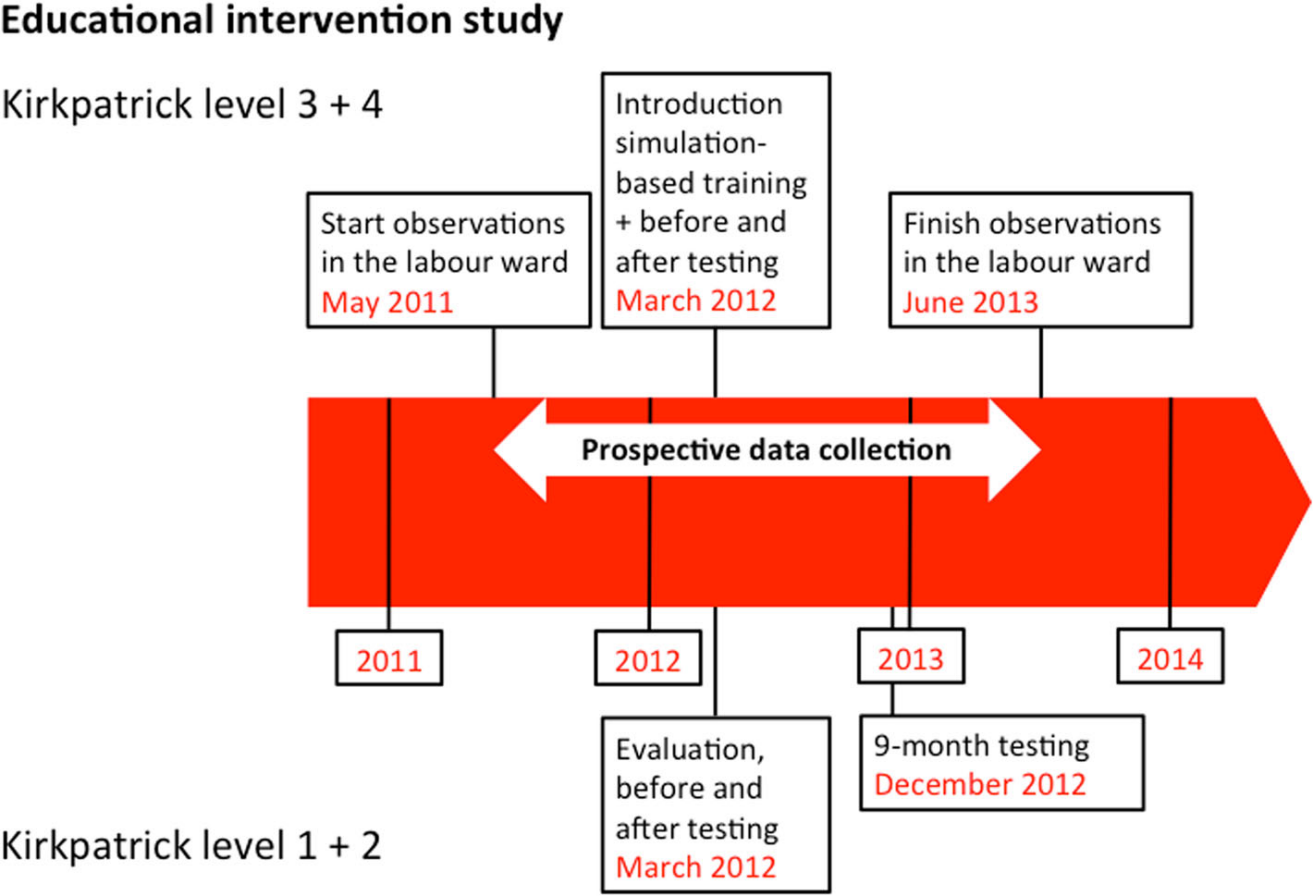
Timeline

The educational intervention took place in March 2012. An overview of research activities is depicted in Fig. 2. Data were prospectively collected from the 25th of May 2011 to the 25th of June 2013. A multi-professional group of clinicians, nurse-midwives, medical attendants (nurse aides without formal medical education), and ambulance drivers (without formal education) attended a half-day training course "Helping Mothers Survive Bleeding After Birth”. The training was conducted in small groups of maximum six participants per facilitator, and consisted of a mix of theory and hands-on practice using a low-cost low-tech simulator (MamaNatalie, Laerdal Global Health). The learning goals were to be able to independently manage an uncomplicated delivery, including active management of third stage of labour by giving 10 IU of oxytocin within one minute after birth, and management of postpartum haemorrhage including bimanual uterine compression. Further details of the intervention and the evaluation of the training are described elsewhere. [8]

### Data measurement

Sixteen research assistants were trained by the principal investigator to structurally observe all births beyond a gestational age of 28 weeks in the hospital. To ensure interrater reliability, the research assistants had a refresher training every three months. Subsequently, their observation skills were assessed in simulated scenarios and on labour ward. Any apparent deviations in observations were discussed and corrected immediately. The observations took place in a structured manner using a validated data collection form with accompanying standard operating procedures. The following background information was recorded for each birth: antenatal care attendance by checking antenatal cards, gestational age at delivery by measuring fundal symphysis height prior to delivery by the birth attendant (as no 12-week dating scan available), mode of delivery (direct observation), birth weight (direct observation), and training attendance including HMS BAB training (by asking the birth attendant). Furthermore, the primary outcome measure was recorded: incidence of postpartum haemorrhage. Blood loss was measured as was usual practice in Haydom Lutheran Hospital and comprised visual estimation, measurement using scales, or a mix of both. The method of measuring blood loss was also recorded. Postpartum haemorrhage was defined as blood loss of 500 ml or more. Severe postpartum haemorrhage was defined as blood loss of 1000 ml or more. Secondary outcome measures were direct observations and included: use of uterotonic drugs after birth (categorized as: oxytocin 10 IU, oxytocin 5 IU, none, and other uterotonics such as misoprostol or other dosages of oxytocin), timing of administration of uterotonic drugs, removal of placenta by controlled cord traction, uterine massage, and examination of the placenta. In cases complicated by postpartum haemorrhage, its management was observed: use of uterotonic drugs in addition to those given routinely after birth (categorized as: oxytocin 10 IU, ergometrine 0.2 mg, none, and other uterotonics such as misoprostol or other dosages of oxytocin), uterine massage, examination of perineum, vagina, and/or episiotomy, bimanual uterine compression, hysterectomy, and units of blood received within 24 h. Lastly, maternal and neonatal outcome at 24 h were recorded. A research supervisor reviewed the completed data collection forms regularly. In case of missing information or discrepancies, the data collection form was returned to the research assistant to be completed in accordance with the original hospital notes under supervision of the research supervisor.

### Sample size consideration

In peer-reviewed articles, incidence rates of postpartum haemorrhage range between 2 and 26%, depending on the definition of postpartum haemorrhage and the method of measuring blood loss. [2, 3, 24, 25] Based on these data, we hypothesized an incidence of postpartum haemorrhage (defined as blood loss of 500 ml or more) of 10%. To show a 25% decrease in the incidence of postpartum haemorrhage with 80% power and a confidence interval of 95%, a sample of 2010 births before, and the same number of births after intervention was needed. If the incidence of postpartum haemorrhage would lie in the lower range cited in the literature (2%) a sample of 11,153 births would be needed before and after the intervention. If the incidence would be around the upper estimates (26%), the sample size needed would be 656 births before and after intervention.

### Statistical analysis

Data were entered twice by two different data clerks and crosschecked in EpiData (The EpiData Association, Odense, Denmark). We used IBM SPSS Statistics (version 20) for data-analysis (IBM, Armonk, NY, USA). In order to assess the effect of the intervention, the study period was divided in two: 25th May 2011 to 6th March 2012 (before training) and 13th March 2012 to 25th June 2013 (after training). The training course took place from the 7th to the 12th of March 2012 and this period was excluded from the analysis. Observations before and after training were compared. Descriptive statistics were calculated for the background characteristics of women who delivered during the study period, the clinical performance of healthcare workers attending births (basic delivery skills and management of postpartum haemorrhage), and patient outcome. Results are reported in numbers (n) and proportions (%) for categorical variables, and mean and standard deviation (SD) for continuous variables. The chi-square test was used for comparison of categorical variables. If numbers were smaller than five, the fisher exact test was used. The independent samples t-test (2-sided) was used to compare continuous variables.

## Results

There were 9446 births observed during the study period from May 2011 to June 2013, 3622 births before and 5824 births after intervention. Ten clinicians were working at the hospital and eight of them attended training (80%). Of the 25 nurse-midwives working in labour ward, 15 attended training (60%), and 14 out of 19 medical attendants attended training (74%). Six out of ten ambulance drivers were trained (60%). On average 54.1% of all births were attended by a HMS BAB trained health care worker. To look at staff turnover, the period after training was divided in three-month periods. In the first three months after training 31.9% of the births were attended by a HMS BAB trained health care worker. In the second three-month period this proportion was 68.8%, then 57.7%, 62.9%, and 48.1% in the consecutive three-month time periods.

Table 1 summarizes the baseline characteristics. More than 97% of the women attended antenatal care. This remained constant throughout the entire study period. Women giving birth after training had a slightly higher mean gestational age (36.4 weeks) compared to women giving birth before training (36.2 weeks) (*p* < 0.001). Most babies were delivered vaginally (before training 82.4%, after training 81.0%). After training more caesarean sections were done (16.1%) compared to before training (13.5%) (*p* < 0.001). Babies born before training had a higher mean birth weight (3107 g) compared to babies born after training (3074 g) (*p* = 0.001).

**Table 1:** Baseline characteristics

	Before training (*n* = 3622)	After training (*n* = 5824)	*P* value
Antenatal care attendance, n (%)	3530 (97.5)	5684 (97.6)	0.46
Gestational age, mean (SD), weeks^a^	36.2 (1.1)	36.4 (1.3)	<0.001
Mode of delivery, n (%)			
Vaginal delivery	2984 (82.4)	4717 (81.0)	0.07
Breech delivery	66 (1.8)	51 (0.9)	<0.001
Assisted vaginal delivery	26 (0.7)	25 (0.4)	0.06
Caesarean section	488 (13.5)	940 (16.1)	<0.001
Missing	58 (1.6)	91 (1.6)	
Birth weight, mean (SD), gram	3107 (474)	3074 (473)	0.001
Births attended by HMS BAB trained person, n (%)	0 (0)	3148 (54.1)	**

^a^In most women there was no reliable estimated date of delivery as calculation by last menstrual period is uncertain and no dating scan facilities are available. Therefore, gestational age was calculated by measuring the fundal symphysis height prior to delivery

**Unable to compute *P* value as before training no one was trained

Table 2 shows patient outcome before and after training. Among the births observed (*n* = 9446) there was a statistically significant difference in blood loss between before (mean 216 ml blood loss, SD 130 ml) and after intervention (mean 207 ml blood loss, SD 130 ml) (p = 0.001). However, Cohen’s effect size value (d = 0.07) suggested small practical relevance. After training there was a significant reduction in the incidence of postpartum haemorrhage (500–1000 ml) from 2.1% to 1.3% (*p* = 0.003). No difference was seen in the group with blood loss more than 1000 ml (before training 0.4%, after training 0.4%). In most cases blood loss was estimated visually (before training 89.3%, after training 90.8%). The number of maternal deaths within 24 h after birth did not change after training (*n* = 1 before training versus *n* = 2 after training). None of the maternal deaths were attributed to postpartum haemorrhage. Neonatal mortality did not change after training.

**Table 2:** Incidence of postpartum haemorrhage and patient outcome before and after intervention

	Before training, n (%) (n = 3622)	After training, n (%) (n = 5824)	*P* value
Blood loss			
< 500 ml	3529 (97.4)	5721 (98.2)	0.008
500–1000 ml	77 (2.1)	78 (1.3)	0.003
≥ 1000 ml	16 (0.4)	25 (0.4)	0.93
Method of estimating blood loss			
Visual	3236 (89.3)	5286 (90.8)	0.02
Measured	165 (4.6)	122 (2.1)	<.001
Both	221 (6.1)	416 (7.1)	0.05
Maternal outcome after 24 h			
Admitted to MW, discharged <24 h	1274 (35.2)	1594 (27.4)	<.001
Admitted to MW, discharged >24 h	2331 (64.4)	4201 (72.1)	<.001
Admitted to ICU < 24 h	16 (0.4)	27 (0.5)	0.88
Death <24 h	1 (0.03)	2 (0.03)	0.86
Perinatal outcome after 24 h			
Normal	3423 (94.5)	5494 (94.3)	<.001
Any kind of difficulties	11 (0.3)	58 (1.0)	<.001
Died after birth	29 (0.8)	50 (0.9)	0.84
Stillbirth (fresh)	58 (1.6)	68 (1.2)	0.07
Stillbirth (macerated)	43 (1.2)	72 (1.2)	0.84
Missing	58 (1.6)	82 (1.4)	

*MW* Maternity Ward, *ICU* Intensive Care Unit

Table 3 shows the clinical performance of basic delivery skills before and after training. The proportion of women receiving 10 IU of oxytocin after training (91.7%) was significantly higher compared to before training (87.8%). There was no difference in the proportion of women that did not receive uterotonic drugs after delivery (before training 3.0%, after training 2.9%). After training, a significantly greater proportion of women received uterotonic drugs within one minute after birth (before training 40.4%, after training 44.3%). Removal of placenta by controlled cord traction and subsequent uterine massage were more frequently performed after training (98.8% and 99.0% respectively) compared to before training (96.5% and 93.0% respectively). There was no difference in the proportion of women that had their placenta examined for completeness after delivery (before training 36.9%, after training 37.0%).

**Table 3:** Basic delivery skills

	Before training, n (%) (n = 3622)	After training, n (%) (n = 5824)	*P* value
Uterotonic drugs			
Oxytocin, 10 IU	3180 (87.8)	5338 (91.7)	<.001
Oxytocin, 5 IU	69 (1.9)	138 (2.4)	0.14
None	108 (3.0)	170 (2.9)	0.83
Other	247 (6.8)	178 (3.1)	<.001
Missing	18 (0.5)	0 (0)	
Uterotonic drugs administered within one minute after birth	1465 (40.4)	2578 (44.3)	0.001
Missing	139 (3.8)	176 (3.0)	
Removal of placenta by controlled cord traction	3494 (96.5)	5757 (98.8)	<.001
Missing	2 (0.1)	0 (0)	
Uterine massage	3367 (93.0)	5767 (99.0)	<.001
Missing	4 (0.1)	0 (0)	
Examination of placenta	1338 (36.9)	2157 (37.0)	0.96
Missing	4 (0.1)	0 (0)	

Table 4 shows management of postpartum haemorrhage before and after training. In the entire study period, 196 women (2.1%) experienced postpartum haemorrhage of 500 ml blood loss or more. The proportion of women with postpartum haemorrhage receiving 10 IU of oxytocin as part of management of postpartum haemorrhage significantly increased from 43.0% before training to 61.2% after training. The proportion of women with postpartum haemorrhage that did not receive any uterotonic drugs increased from 1.1% before training to 3.9% after training, but this was not statistically significant. After training, more women received uterine massage (before 80.6%, after 90.3%), examination of perineum, vagina, and/or episiotomy (before 51.6%, after 64.1%), and bimanual uterine compression (before training 11.8%, after training 19.4%) as part of the management of postpartum haemorrhage. However, these changes were not statistically significant. The proportion of women having a hysterectomy after postpartum haemorrhage remained constant at around 3%. The number of blood units given within 24 h to women experiencing postpartum haemorrhage increased slightly from an average of 0.4 units before training to an average of 0.7 units after training.

**Table 4:** Management of postpartum haemorrhage

	Before training, n (%) *n* = 93	After training, n (%) *n* = 103	*P* value
Uterotonic drugs			
Oxytocin, 10 IU	40 (43.0)	63 (61.2)	0.04
Ergometrine, 0.2 mg	12 (12.9)	18 (17.5)	0.49
None	1 (1.1)	4 (3.9)	0.38
Other	34 (36.6)	18 (17.5)	0.001
Missing	6 (6.5)	0 (0)	
Uterine massage	75 (80.6)	93 (90.3)	0.19
Missing	6 (6.5)	2 (1.9)	
Examination of perineum, vagina, episiotomy	48 (51.6)	66 (64.1)	0.16
Missing	6 (6.5)	2 (1.9)	
Bimanual uterine compression	11 (11.8)	20 (19.4)	0.22
Missing	7 (7.5)	0 (0)	
Hysterectomy	3 (3.2)	3 (2.9)	0.99
Missing	7 (7.5)	0 (0)	
Units of blood given <24 h			
No blood given	56 (60.2)	59 (57.3)	0.26
1 unit	23 (24.7)	28 (27.2)	0.95
≥ 2 units	6 (6.5)	15 (14.6)	0.10
Missing	8 (8.6)	1 (0.9)	

## Discussion

### Main findings

The incidence of postpartum haemorrhage before the intervention was near the lower limits of the range between 2% and 26% found in the literature. [2, 3, 24, 25] Nevertheless, our study showed a 38% reduction (from 2.1% before to 1.3% after training) in the incidence of postpartum haemorrhage (500–1000 ml) following the introduction of the HMS BAB training programme. However, the effect size was low and therefore of small clinical significance, mainly because of the already low incidence before the intervention. The reduction in postpartum haemorrhage was associated with an improvement in clinical performance of basic delivery skills and management of postpartum haemorrhage. The latter was not statistically significant except for an increase in the use of oxytocin for treatment of postpartum haemorrhage. These results are highly relevant considering the high prevalence of anaemia in pregnancy in this population and the fact that most of these settings do not have blood banks. [26]

### Strengths and limitations

The improvements seen after the introduction of the training course may be caused by other changes in the healthcare system. A parallel study on the effect of simulation training in neonatal resuscitation took place around the same time as this study. In addition, the labour ward was expanded during the time of the study. However, we do not think these changes had any influence on the outcomes studied in this project, as it has not changed practice regarding the prevention and management of postpartum haemorrhage. No new guidelines, protocols, or obstetric initiatives were introduced in the hospital during the study period. Only one person attended an external emergency obstetric care training course. In addition, we have evaluated the HMS BAB programme on all four levels of the Kirkpatrick model. [8, 27] Because there is improvement across most levels of the Kirkpatrick model, we consider it very likely that the reduction in incidence of postpartum haemorrhage and improvement in clinical performance was due to the training course.

It was not feasible to power our study to investigate the effect of training on maternal mortality. Based on a cross-sectional study that took place in Haydom Lutheran Hospital from 2009 to 2011, [26] we would expect approximately 16 maternal deaths and maternal near misses related to postpartum haemorrhage (prevalence 0.64%) per year. A sample of approximately 70,000 births would be needed to show a reduction of 25% in postpartum haemorrhage related maternal mortality and severe maternal morbidity.

Gestational age was estimated by measuring the fundal symphysis height. This is most likely not very accurate as the mean birth weight in this cohort was around 3100 g and the mean gestational age was 36 weeks. However, this method is common practice in most low-resource settings [28] and was used consistently throughout the study period. Therefore, both periods before and after intervention have the same measurement flaw. We have accepted this because of unreliable date of last menstrual period and lack of dating scan possibilities.

Lastly, the presence of research assistants may have influenced the performance of the healthcare workers being observed (Hawthorne effect). We expect this effect to be minimal as the research assistants had been present in labour ward for more than two years, collecting data by observing births for other studies, and were considered part of the team working in labour ward. [29–31]

### Interpretation

The overall incidence of postpartum haemorrhage was almost 8% lower than expected. Sheldon et al. describe a similar finding in the WHO Multicountry Survey on Maternal and Newborn Health. [32] The measured incidence is most likely to be an underestimation of the actual incidence because in the majority of women blood loss was estimated visually. It is known that subjective measurement of blood loss leads to lower estimates of the incidence of postpartum haemorrhage. [3, 33] Furthermore, in our cohort the proportion of women with postpartum haemorrhage receiving bimanual uterine compression and hysterectomy was relatively high, indicating severe bleeding. To objectively measure blood loss, we explored the use of point-of-care Hb measurements before and after birth and the use of blood collection bags. However, this did not fit our research budget and the idea met resistance from nurse midwives who already experienced a high workload. The likely underestimation of blood loss influences the study power. In the group of women with more than 500 ml blood loss after birth, an improvement in management of postpartum haemorrhage was observed. However, because of the lower than expected incidence of postpartum haemorrhage, our sample size was too small to show a statistically significant difference. It is important to emphasize the need for objective blood loss measurement in future research in low-resource settings. It will not only improve the reliability of the outcomes, but may also improve quality of care in general by better detection of major haemorrhage and subsequent management.

A significant increase in caesarean sections was observed after training. Performing caesarean sections was not addressed during training. We do not know the reason for this increase, other than a reflection of the global increase in caesarean sections, but we know caesarean sections are associated with an increased amount of blood loss after birth compared to vaginal births. [34] This increase in blood loss associated with caesarean sections was not seen after training. Instead a reduction of blood loss was seen. The reduction in postpartum haemorrhage may have been more pronounced if the caesarean section rate would have remained stable.

The results of this study are very relevant for a population in which 76% of women suffer from anaemia in pregnancy. [26] In addition, in many low-resource settings such as Haydom Lutheran Hospital, there is no blood bank and women depend on their relatives for blood for transfusion. A reduction of haemorrhage in the group with 500–1000 ml blood loss, was assumed to be possible due to improvement in the basic steps of management of postpartum haemorrhage such as giving oxytocin, removing the placenta by controlled cord traction, and performing uterine massage. The mannequin that was used in the HMS BAB training programme has features that very realistically simulate these basic steps: a placenta that can be removed by controlled cord traction, a uterus that can contract and relax, and the ability of the mannequin to bleed up to 1500 ml. These features may have helped the learning of these skills. However, in women with severe blood loss after birth (> 1000 ml) the basic steps may not have been sufficient enough to control bleeding caused by uterine atony, and certainly not for other causes of postpartum haemorrhage. Therefore, we were not able to observe a reduction in severe postpartum haemorrhage.

HMS BAB was also introduced and evaluated in India, Malawi, and Zanzibar in 2012. [13] This study reported on good acceptability and increase in knowledge and skills after introduction of the training programme, however, it did not report on effect on clinical performance and patient outcome. Currently, the HMS BAB programme is being evaluated in Uganda.

Other studies investigating the impact of obstetric emergency training report mostly on neonatal outcome, and only few studies report on maternal outcome. [16, 17] The QUARITE study showed a reduction in maternal mortality after a multifaceted intervention to promote maternity death reviews and onsite training in emergency obstetric care. [20] Two further studies show a decrease in postpartum haemorrhage after introduction of an obstetric training programme. [18, 21] Sorensen et al. studied the impact of the Advanced Life Support in Obstetrics (ALSO) course in Tanzania and found no difference in the management of prolonged labour and neonatal care. [35] There was, however, a significant improvement in active management of third stage of labour and management of postpartum haemorrhage after training. [18] The above studies reinforce the results of our study that simulation-based training can be used to improve clinical performance and patient outcome.

## Conclusions

A half-day obstetric simulation-based training HMS BAB was associated with a reduction in the incidence of postpartum haemorrhage (500–1000 ml). Clinical performance of basic delivery skills and treatment of postpartum haemorrhage with oxytocin increased significantly after training. Other skills in management of postpartum haemorrhage (uterine massage, examination of birth canal, and bimanual uterine compression) improved after training, but this was not statistically significant. The study power has suffered from the much lower than anticipated incidence of postpartum haemorrhage. Future research should focus on objective measurement of blood loss and consider using a randomised study design.

## Abbreviations

HMS BAB: Helping Mothers Survive Bleeding After Birth
n: number
PPH: Postpartum Haemorrhage
SD: Standard Deviation
